# Tumor genomics in patients younger than 40 years of age with metastatic breast cancer

**DOI:** 10.1038/s41698-026-01333-0

**Published:** 2026-02-26

**Authors:** Kristen D. Brantley, Ananya Kodali, Gregory J. Kirkner, Melissa E. Hughes, Yvonne Li, Janet Files, Sarah Strauss, Anne-Marie Feeney, Ayesha Mohammed-Abreu, Romualdo Barroso Sousa, Brittany Bychkovsky, Charlotte Tannenbaum, Maggie Loucks, Barbara K. LeStage, Tari King, Bruce E. Johnson, Lynette Sholl, Deborah Dillon, Sara M. Tolaney, Andrew D. Cherniack, Ann H. Partridge, Nancy U. Lin, Ana C. Garrido-Castro

**Affiliations:** 1 Department of Medical Oncology, Dana-Farber Cancer Institute, Boston, MA USA; 2 Breast Oncology Program, Dana-Farber Brigham Cancer Center, Boston, MA USA; 3 Dasa Institute for Education and Research (IEPD), Brasilia, Brazil; 4 Dasa Oncology/Hospital Brasilia, Brasilia, Brazil; 5 Division of Cancer Genetics and Prevention, Dana-Farber Cancer Institute, Boston, MA USA; 6 Harvard Medical School, Boston, MA USA; 7 Division of Breast Surgery, Department of Surgery, Brigham and Women’s Hospital, Boston, MA USA; 8 Department of Pathology, Brigham and Women’s Hospital, Boston, MA USA; 9 Broad Institute of Harvard and MIT, Cambridge, MA USA; 10 Present Address: Yale Cancer Center, New Haven, CT USA; 11 Present Address: Winship Cancer Institute of Emory University, Atlanta, GA USA

**Keywords:** Biomarkers, Cancer, Genetics, Oncology

## Abstract

Unique disease characteristics of younger patients warrants investigation of tumor genomics in young-onset metastatic breast cancer (MBC). Targeted DNA sequencing was completed for tumors of MBC patients diagnosed between 2009-2020. Multivariable logistic regression tested associations between single nucleotide variants (SNVs) and copy number variants and age at MBC diagnosis. Multivariable Cox regression estimated hazard ratios for overall survival (OS) by somatic alterations. Among 2,357 MBC patients, tumors of those ≤40 years at diagnosis (vs. >55) were more likely to harbor amplifications in *ERBB2* and *MYC* (p < 0.01) and mutations in *TP53* (odds ratio [OR] = 1.83, p < 0.001), and less likely to harbor mutations in *CDH1* and *PIK3CA* (p < 0.001). OS was shorter among younger recurrent MBC patients [median: 2.8 (≤ 40) vs. 3.6 years ( > 55), p = 0.04], with SNVs in *TP53* and *PTEN* associated with shorter OS. Distinct tumor genomics of young-onset MBC patients suggest differences in tumor biology that should guide investigation of targetable pathways.

## Introduction

While recent treatment advances have successfully extended the lifespans of patients diagnosed with metastatic breast cancer (MBC)^1^, MBC remains an incurable disease with a brief survival period dependent on molecular subtype, estimated to range from 1.6 years for hormone receptor-negative/HER2-negative (HR-/HER2-) tumors to 4.9 years for hormone receptor-positive/HER2-negative (HR + /HER2-) tumors^2^. Recent increases in rates of MBC among young women highlight the importance of studying the tumor characteristics in this age group. From 2004-2021, the incidence of MBC at initial presentation (de novo, dnMBC) increased most dramatically among women under 40 years of age at diagnosis^3^. In recent years we have also seen an increase in the rates of early-stage breast cancer among women under 40 years of age^4,5^. Younger women with early-stage breast cancer are typically diagnosed with more aggressive tumors, increasing their risk of developing recurrent MBC (rMBC)^6–8^. A recent meta-analysis of 280,000 patients found pooled proportions of metastatic recurrence to be highest (approximately 18.6%) among those <50 years at initial diagnosis with early-stage breast cancer^9^.

In addition to the rising incidence rates, several population-based studies have noted key survival differences for MBC patients based on age which are not fully explained by clinical factors. For instance, younger dnMBC patients tend to survive longer than their older counterparts, even after accounting for patient and tumor characteristics^10–12^. However, younger age does not confer the same survival advantage among rMBC patients diagnosed within five years of primary diagnosis^13,14^. As the underlying reasons for these age-specific survival differences remain elusive, it is of great interest to comprehensively characterize MBC tumors by age.

Tumor genomic profiling has proven useful in developing and deploying targeting therapies for MBC patients^15–18^. The genomic profile provides an avenue for better defining the etiology and progression of MBC based on age at diagnosis. A recent study within our group showed distinct genomic profiles comparing the oldest group of MBC patients ( ≥ 70 years at diagnosis) to those <55 years at diagnosis^19^. Studies of early-stage breast cancer patient cohorts have shown that somatic alterations are different among women diagnosed at ≤40 years compared to those diagnosed at more advanced ages^20–22^, even within the same intrinsic molecular subtypes. Whether genomic differences exist among these young MBC patients remains to be seen. Here we leverage a large, well-annotated clinical dataset to uncover differences in genomic profiles of MBC patients comparing the youngest patients (≤ 40 years at MBC diagnosis) with those 40-55 and >55 years at diagnosis. It is additionally important to evaluate tumor genomics of those ≤40 years separately from other ages given that this age group represents women who, for the most part, have not begun to receive regular screening mammograms per the U.S. recommendations^23^. We also sought to evaluate survival differences based on the combination of age and observed tumor genomic characteristics.

## Results

### Patient and Tumor Characteristics

Between July 2013-December 2020, 2369 women diagnosed with MBC within the Ending Metastatic Breast Cancer for Everyone (EMBRACE) cohort had tumor samples sequenced using OncoPanel and provided consent. After excluding 12 patients with missing age at MBC diagnosis, the cohort included 2357 women, of which 320 were diagnosed with MBC at 40 years or younger, 929 were diagnosed at age 41–55 years, and 1108 were diagnosed after age 55 (Table 1). Most participants were White (87%) and had HR + /HER2- disease (57%). Luminal A and luminal B subtypes were inferred among HR + /HER2- tumors based on grade and PR staining (luminal A-like: primary tumor grade <3 and metastatic tumor PR staining ≥10%; luminal B-like: primary tumor grade=3 or metastatic tumor PR staining <10%). Luminal B-like tumors accounted for a greater proportion of HR + /HER2- tumors in younger women (≤ 40 years: 70% vs. >55 years: 50%). Overall, MBC tumors of younger women were more likely to be HER2+ (≤ 40 years 30% vs. >55 years 13%) or triple-negative breast cancer (TNBC, ≤40 years 21% vs. >55 years 16%). A total of 607 (26%) individuals were diagnosed with dnMBC, which was more common in the youngest age group (≤ 40 years 37% v. 41–55 years 25% vs. >55 years 24%). Tumor sequencing and molecular subtyping were performed on 766 (33%) primary tumors and 1591 (68%) metastatic tumors, with similar distributions of sample type in each age group. Of the 1475 (63%) patients who had available germline mutation test results at Dana-Farber Cancer Institute (DFCI), 110 (7%) had a known pathogenic or likely pathogenic (P/LP) germline variant. The most common germline P/LP variants were *BRCA1* (0.9%), *BRCA2* (2.0%), *CHEK2* (1.2%), and *PALB2* (0.9%). The most common metastatic sites aside from breast were liver (*N* = 361, 23%) and bone (*N* = 175, 11%) (Table S1). Median follow-up from time of metastatic diagnosis was 5.6 years (95% CI = 5.3–6.0 years). Approximately 58% of patients had died at last follow-up and proportions did not differ by age group at diagnosis (58% if ≤40 years, 60% if 40-55 years, 57% if >55 years).

**Table 1:** Demographic and Tumor Characteristics of Patients in EMBRACE with OncoPanel Sequencing (*N* = 2357)

		Age group at metastatic dx		
	All ages	<=40 y (*N* = 320)	41-55 y (*N* = 929)	>55 y (*N* = 1,108)
Variable	N (%) or Mean (SD)	N (%) or Mean (SD)	N (%) or Mean (SD)	N (%) or Mean (SD)
Age at primary diagnosis (y)	49.9 (11.5)	33.2 (4.1)	45.2 (5.2)	58.7 (8.9)
Age at metastatic diagnosis (y)	54.1 (11.9)	34.8 (4.0)	48.5 (4.3)	64.3 (6.8)
Race				
White	2057 (87%)	267 (83%)	795 (86%)	995 (90%)
Black	110 (4.7%)	17 (5.3%)	47 (5.1%)	46 (4.2%)
Asian	85 (3.6%)	19 (5.9%)	39 (4.2%)	27 (2.4%)
Other	51 (2.2%)	13 (4.1%)	22 (2.4%)	16 (1.4%)
Unknown	54 (2.3%)	4 (1.3%)	26 (2.8%)	24 (2.2%)
Germline genetic testing done	1,475 (63%)	207 (65%)	566 (61%)	702 (63%)
Any P/LPV (if tested)^a^	110 (7.5%)	20 (9.7%)	48 (8.5%)	42 (6.0%)
* BRCA1* P/LPV (if tested)^a^	14 (0.9%)	4 (1.9%)	5 (0.9%)	5 (0.7%)
* BRCA2* P/LPV (if tested) ^a^	30 (2.0%)	4 (1.9%)	16 (2.8%)	10 (1.4%)
* PALB2* P/LPV (if tested) ^a^	14 (0.9%)	3 (1.4%)	5 (0.9%)	6 (0.9%)
* CHEK2* P/LPV (if tested) ^a^	17 (1.2%)	2 (1.0%)	8 (1.4%)	7 (1.0%)
Primary tumor stage				
Stage 0 (in situ)	29 (1.2%)	3 (0.9%)	14 (1.5%)	12 (1.1%)
Stage 1	334 (14%)	23 (7.2%)	119 (13%)	192 (17%)
Stage 2	781 (33%)	91 (28%)	331 (36%)	359 (32%)
Stage 3	583 (24%)	83 (26%)	232 (25%)	268 (24%)
Stage 4 (de novo metastatic)	607 (26%)	119 (37%)	228 (25%)	260 (24%)
Missing	23 (1%)	1 (0.3%)	5 (0.5%)	17 (1.5%)
Primary tumor grade				
1	136 (5.8%)	5 (1.6%)	52 (5.6%)	79 (7.1%)
2	908 (39%)	78 (24%)	354 (38%)	476 (43%)
3	1,161 (49%)	228 (71%)	477 (51%)	456(41%)
Missing	152 (6.4%)	9 (2.8%)	46 (4.9%)	97 (8.8%)
Primary tumor subtype				
Luminal A-like^b^	668 (28%)	46 (14%)	293 (32%)	329 (30%)
Luminal B-like^c^	682 (29%)	109 (34%)	240 (26%)	333 (30%)
HR + /HER2+	243 (10%)	58 (18%)	99 (11%)	86 (7.8%)
HR-/HER2+	165 (7%)	37 (12%)	69 (7.4%)	59 (5.3%)
HR-/HER2-	425 (18%)	66 (21%)	178 (19%)	181 (16%)
Missing	174 (7%)	4 (1.3%)	50 (5.4%)	120 (11%)
Time from primary BC to met				
De novo	607 (26%)	119 (37%)	228 (25%)	260 (24%)
>0-24 months	470 (19%)	93 (29%)	213 (23%)	164 (15%)
>=24 months	1279 (54%)	108 (34%)	488 (53%)	683 (62%)
Metastatic tumor subtype^d^				
Luminal A-like^b^	673 (29%)	51 (16%)	262 (28%)	360 (33%)
Luminal B-like^c^	730 (31%)	101 (32%)	262 (28%)	367 (33%)
HR + /HER2+	212 (9%)	51 (16%)	93 (10%)	68 (6.1%)
HR-/HER2+	184 (7.8%)	37 (12%)	73 (7.9%)	74 (6.7%)
HR-/HER2-	531 (23%)	79 (25%)	226 (24%)	226 (20%)
Missing	27 (1.1%)	1 (0.3%)	13 (1.4%)	13 (1.2%)
Sample Type Sequenced				
Primary	698 (30%)	120 (38%)	279 (30%)	299 (27%)
Locoregional	68 (2.9%)	2 (0.6%)	23 (2.5%)	43 (3.9%)
Metastatic	1591 (68%)	198 (62%)	627 (68%)	766 (69%)
Died	1377 (58%)	184 (58%)	558 (60%)	635 (57%)
Time from met dx to death or censorship (y)	3.4 (3.1)	3.3 (3.1)	3.6 (3.5)	3.3 (2.8)

*HR* hormone receptor, *y* years, *P/LPV* pathogenic/likely pathogenic variant.

^a^Percent calculated out of total tested for any germline variant (*N* = 1475 total).

^b^HR + /HER2- with primary tumor grade <3 & met tumor PR staining >=10%.

^c^HR + /HER2- with primary tumor grade=3 OR met tumor PR staining <10%.

^d^Determined using primary subtype if met. subtype not available (*N* = 527).

### Tumor Mutational Burden

**Fig. 1:**
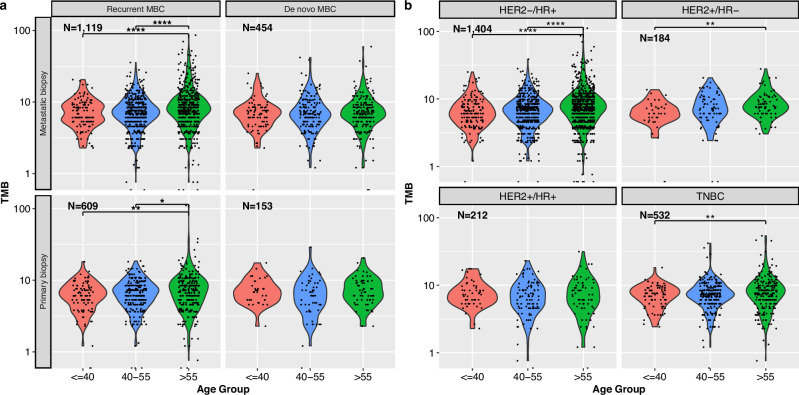
Tumor mutational burden by age at MBC diagnosis. **a** Type of Sequenced Sample (either primary tumor or metastatic tumor) and stage at initial diagnosis and **b** tumor subtype. p-values: *: *p* < 0.05, **: *p* < 0.01, ***: *p* < 0.001, ****: *p* < 0.0001.

Tumor mutational burden (TMB) was higher among those older at metastatic diagnosis for patients experiencing rMBC (*p* < 0.0001), though no statistically significant differences were observed for patients with dnMBC (Fig. 1a). Similar differences in TMB by age were observed when assessing within individual tumor subtypes, though the association was strongest among patients with HR + /HER2- MBC (*p* < 0.0001) (Fig. 1b). Further examination within this group revealed the age-TMB association was only statistically significant among those with luminal B-like tumors (Fig. S1).

### Genetic Alterations by Age Group at Diagnosis and Molecular Subtype

**Fig. 2:**
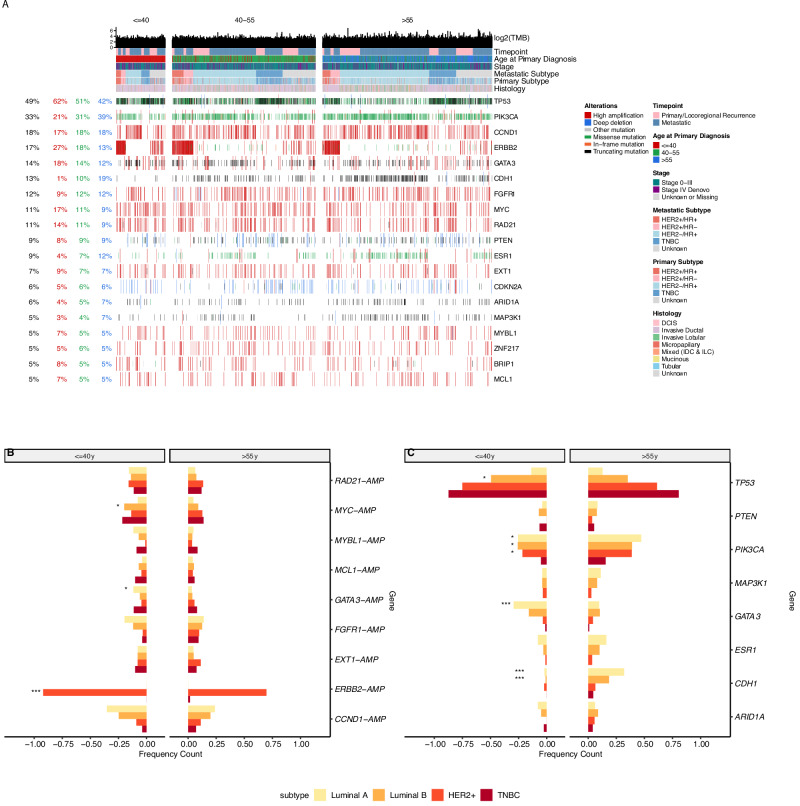
Genomic alterations by age at diagnosis of MBC. Alterations were detected in metastatic (where available) or primary tumors for individuals with MBC by tumor subtype and age at diagnosis. **A** Oncoprint shows gene alteration frequency for select genes ( ≥ 5% altered in entire population), grouped by age at metastatic diagnosis (<=40 y, 40–55 y, >55 y). Annotations on left hand side display percent alterations per gene overall (in black), among those <=40 y (red), among those 40–55 y (green) and among those >55 y (blue). **B** Frequency of CNV alterations by age at MBC diagnosis (<=40 y vs. >55 y). **C** Frequency of SNV alterations by age at MBC diagnosis (<=40 y vs. >55 y). For (**B**) and (**C**) genes are displayed if alterations occurred at ≥5% in the entire population and if mutation was present in at least two tumor molecular subtypes. Statistical tests of comparison are Fisher exact tests, comparing individuals ≤40 y vs. >55 y at diagnosis. *: *p* < 0.05, **: *p* < 0.01, ***: *p* < 0.001.

Several differences in gene alteration frequency (including both copy number variants [CNVs] and single nucleotide variants [SNVs]) were apparent by age group at diagnosis within individual tumor subtypes (Fig. 2).

Assessment of CNVs alone revealed that amplifications were more common in *MYC* for luminal B-like disease and *GATA3* for luminal A-like disease among those ≤40 years at MBC diagnosis, compared with those >55 years (Fig. 2B). Those ≤40 years at MBC diagnosis with HER2+ disease also had higher frequency of *ERBB2* amplifications, though this was not corrected for aneuploidy.

Several differences in SNV frequency emerged between age groups, with some variation of magnitude and significance by molecular subtype (Fig. 2C). For example, *TP53* mutations were statistically significantly enriched among younger vs. older individuals with luminal B-like disease (50 vs. 35%, p = 0.03), whereas enrichment of *GATA3* mutations among younger individuals was statistically significant for those with luminal A-like disease (29% vs. 10%, *p* < 0.001). Older individuals had a statistically significantly higher frequency of oncogenic or likely oncogenic mutations in *PIK3CA* compared to younger individuals for all subtypes (HER2- luminal A-like ≤40 years vs. 55 years: 25% vs. 47%, *p* = 0.01, HER2- luminal B-like: *p* = 0.03, HER2 + : 26% vs. 39%, *p* = 0.03, TNBC: 5% vs. 15%, *p* = 0.05). Other PI3K pathway genes, AKT1 and PTEN, did not differ in mutational frequency by age for HER2-/HR+ patients (AKT1: 4% v. 5%, *p* = 1, PTEN: 6% v. 8%, *p* = 1). Frequency of *CDH1* mutations was higher among older individuals, though this was only statistically significant in the HER2-/HR+ subtype (1% vs. 25%, *p* < 0.001). After restricting to invasive lobular cases (*N* = 300), a subtype that is more common in the older age group, *CDH1* mutations remained more common for those >55 years compared to those ≤40 years at MBC diagnosis (74% v. 21%, *p* < 0.001). With the exception of *ESR1*, mutational frequencies by age at MBC diagnosis were similar when assessing primary tumor and metastatic tumor samples separately (Fig. S2). Consideration of somatic SNVs occurring at lower population frequencies ( > 1- < 5%) demonstrated that, compared to those >55 years, younger individuals with luminal B-like tumors possessed more *BRCA1* mutations (6% vs. 1%, *p* = 0.03) and fewer *NF1* mutations (0% vs. 3%, *p* = 0.03). No age differences emerged for other low frequency genes overall or within tumor subtypes.

### Oncogenic Cell Signaling Pathways

Alterations in the *TP53*, *PI3K*, and *MYC* pathways (defined by combined CNV and SNVs) were significantly associated with age at MBC diagnosis after accounting for multiple testing in the HR + /HER2- subtype. The *TP53* pathway was more frequently mutated in younger individuals (40 years v. 55 years, q value = 0.01), and the *MYC* and *PI3K* pathways more frequent in the older individuals (*MYC* q = 0.02, *PI3K* q = 0.01). *TP53* pathway differences were driven by the luminal B-like subtype while *PI3K* differences were driven by the luminal A-like subtype. Within HER2+ tumors, only the *RTK-Ras* pathway was significantly associated with younger age (40 years v. 55 years, q = 0.01). No pathway differences were noted by age in TNBC tumors.

### Logistic Regression Analysis

After adjusting for tumor subtype (HER2- luminal A, HER2- luminal B, HR + /HER2 + , HR-/HER2 + , HR-/HER2-), initial stage of breast cancer (de novo v. stage 0-III), histology of primary breast cancer (invasive ductal cancer [IDC], invasive lobular cancer [ILC], mixed, other including ductal carcinoma in situ [DCIS], micropapillary, mucinous, tubular, and unknown), TMB, timepoint of tumor sample used for sequencing (primary v. metastatic tumor), and race (White, Black, Asian, other, unknown), several CNVs and SNVs were associated with age at metastatic diagnosis (Table 2). *ERBB2*-amplifications and *MYC*-amplifications were more prevalent among individuals ≤40 years vs. those >55 years (odds ratio, OR [95% CI] *ERBB2-AMP* = 3.70 [1.87-7.31], *p* < 0.001; OR [95% CI] *MYC-AMP* = 1.57 [1.07–2.27], *p* = 0.02), though only *ERBB2-AMP* remained significant after multiple testing adjustment (p_adj_<0.001). Among SNVs appearing in at least 5% of the overall population, the strongest associations when comparing ≤40 years to >55 years were observed for *CDH1* (OR [95% CI] = 0.08 [0.03-0.26], *p* < 0.001), *PIK3CA* (OR [95% CI] = 0.54 [0.39-0.76], *p* < 0.001), and *TP53* (OR [95% CI] = 1.83 [1.35-2.48], *p* < 0.001), all of which remained statistically significant after multiple-testing correction. Considering SNVs appearing in ≥1% but <5% of the overall population, compared to those >55 years at diagnosis, younger individuals were more likely to possess *BRCA1* (OR [95% CI] = 3.76 [1.82–7.77], *p* < 0.001) and *BRCA2* (OR [95% CI] = 2.75 [1.46–5.19], *p* = 0.002) variants, and less likely to possess *NF1* variants (OR [95% CI] = 0.23 [0.05-0.98], *p* = 0.05), though *NF1* was not significant after multiple testing adjustment. After removing all individuals with any known germline P/LPs (N = 110), associations between somatic mutational frequency and age group at MBC diagnosis remained largely similar for mutations with >5% population frequency (Table S2). Among low-frequency mutations, *BRCA1* and *BRCA2* remained statistically significant by age (OR [95% CI] ≤40 years v. 55 years *BRCA1* = 4.00 [1.81-8.83], *p* < 0.001; *BRCA2* = 3.01 [1.45-6.23], *p* = 0.003).

**Table 2:** Association between presence of oncogenic and likely oncogenic gene alterations and age group at MBC diagnosis (N = 2307)^a^

Gene	Age group (y) at MBC diagnosis^b^	OR (95% CI)^c^	p-value	q-value^d^
***SNVs***				
*CDH1*	<=40	0.08 (0.03–0.26)	2.0e–05***	8.9e–05***
	40–55	0.53 (0.36–0.77)	9.2e–04***	2.4e–03**
*PIK3CA*	<=40	0.54 (0.39–0.76)	2.8e–04***	1.0e–03 **
	40–55	0.84 (0.69–1.04)	ns	ns
*TP53*	<=40	1.83 (1.35–2.48)	1.0e–04***	3.9e–04 ***
	40–55	1.49 (1.20–1.85)	3.6e–04***	9.4e–04 ***
*GATA3*	<=40	1.47 (0.94–2.30)	ns	ns
	40–55	1.38 (0.99–1.92)	ns	ns
*ESR1*	<=40	0.46 (0.21–0.98)	4.4e–02*	ns
	40–55	0.77 (0.53–1.11)	ns	ns
*MAP3K1*	<=40	0.50 (0.24–1.03)	ns	ns
	40–55	0.57 (0.37–0.88)	ns	ns
*PTEN*	<=40	0.72 (0.40–1.33)	ns	ns
	40–55	1.00 (0.69–1.45)	ns	ns
*ARID1A*	<=40	0.76 (0.38–1.49)	ns	ns
	40–55	1.90 (0.59–1.36)	ns	ns
***CNVs***				
*ERBB2–AMP*	<=40	3.70 (1.87–7.31)	1.7e–04***	6.0e–04***
	40–55	2.19 (1.31–3.66)	2.7e–03**	6.8e–03**
*MYC–AMP*	<=40	1.56 (1.07–2.27)	2.0e–02*	ns
	40–55	1.08 (0.80–1.46)	ns	ns
*RAD21–AMP*	<=40	1.40 (0.94–2.07)	ns	ns
	40–55	1.04 (0.76–1.42)	ns	ns
*ZNF217–AMP*	<=40	1.17 (0.64–2.13)	ns	ns
	40–55	1.40 (0.92–2.14)	ns	ns
*GATA3–AMP*	<=40	1.55 (0.93–2.58)	ns	ns
	40–55	0.83 (0.54–1.28)	ns	ns
*MYBL1–AMP*	<=40	1.12 (0.65–1.91)	ns	ns
	40–55	0.87 (0.57–1.31)	ns	ns
*CCND1–AMP*	<=40	1.10 (0.78–1.57)	ns	ns
	40–55	0.97 (0.76–1.24)	ns	ns
*EXT1–AMP*	<=40	1.12 (0.69–1.82)	ns	ns
	40–55	0.99 (0.69–1.42)	ns	ns
*FGFR1–AMP*	<=40	0.68 (0.44–1.06)	ns	ns
	40–55	0.89 (0.67–1.18)	ns	ns
*MCL1–AMP*	<=40	1.46 (0.84–2.51)	ns	ns
	40–55	0.97 (0.63–1.48)	ns	ns

*MBC* metastatic breast cancer, *y* years, *OR* odds ratio, *CI* confidence interval, *SNV* single nucleotide variants, *CNV* copy number variants, *AMP* amplification, *ns* nonsignificant at α = 0.05; * <0.05, ** <0.01, *** <0.001

^a^Genes selected if mutational frequency >5% in total population and if mutation was significantly enriched or depleted (*p* < 0.05) in at least one tumor molecular subtype. N excludes those with unknown tumor subtype (*N* = 26) and unknown stage (*N* = 23) and individual with HER2-/HR+ subtype without luminal classification (*N* = 1).

^b^Reference age group is >55 y at MBC diagnosis.

^c^All models adjusted for tumor subtype (HER2- luminal A, HER2- luminal B, HR + /HER2 + , HR-/HER2 + , HR-/HER2-), histology (DCIS, invasive ductal, invasive lobular, mixed, other (including DCIS, micropapillary, mucinous, tubular), unknown), TMB, initial stage of BC (de novo, Stage 0-III, unknown), timepoint sampled for genetic sequencing (primary v. metastatic tumor) and race (White, Black, Asian, other, unknown).

^d^FDR-adjusted p-value.

### Association Between Genetic Alterations and Survival by Age at MBC Diagnosis

**Fig. 3:**
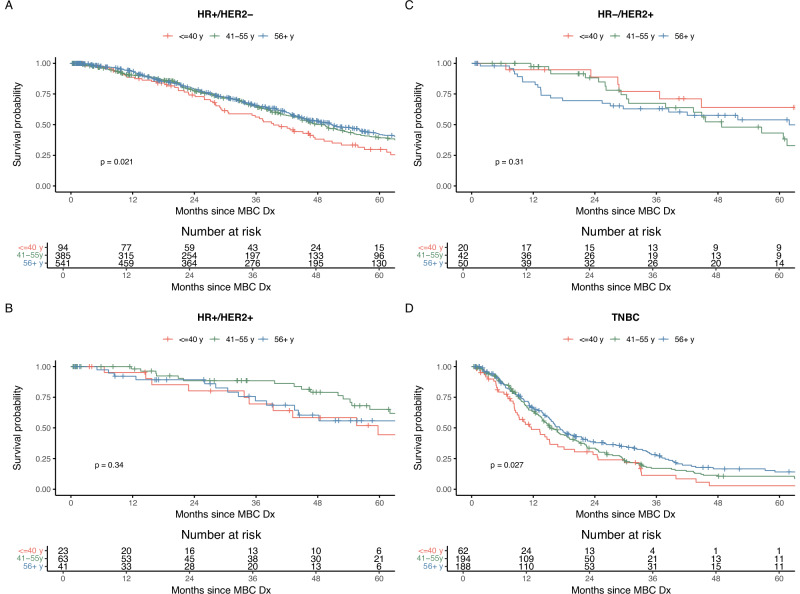
Kaplan-Meier survival curves by age group at metastatic diagnosis. Survival among those with recurrent **A** HR+ /HER2- **B** HR+ /HER2+ **C** HR-/HER2+ , and **D** TNBC MBC (N Total=1710). P-value presented is for the log-rank test between age groups at MBC diagnosis.

Overall, survival differences by age were only observable among individuals who experienced rMBC (Fig. 3), with shorter survival seen among those ≤40 years at MBC diagnosis who had either HR + /HER2- or TNBC subtypes. No age differences were apparent for those with dnMBC (Fig. S3). TMB did not influence survival in those with either dnMBC or rMBC (*p* > 0.05 in crude models).

Amplifications in *ERBB2* were, as expected, associated with prolonged overall survival (OS) (*p* < 0.001) while amplifications in *MYC* and *FGFR1* were associated with shorter OS (p = 0.001 and 0.01, respectively) (Fig. S4). All associations were maintained after adjustment for molecular subtype, TMB, histology, race, stage at initial diagnosis, sample type, age at metastatic diagnosis, number of metastatic sites, and site of metastatic disease (Table 3).

**Table 3:** Hazard Ratios and 95% confidence intervals (CI) for OS by presence vs. absence of oncogenic or likely oncogenic gene alterations, overall and stratified by age at MBC diagnosis (*N* = 2252, Deaths= 1313)

		Age group at MBC diagnosis		
		≤40 y	40-55 y	>55 y
Gene	All age groups	HR (95% CI)	HR (95% CI)	HR (95% CI)
***SNVs***				
*CDH1*				
Model 1^a^	–	0.60 (0.07–5.30)	0.76 (0.49–1.16)	1.10 (0.81–1.50)
Model 2^b^	1.00 (0.79–1.27)	0.64 (0.07–5.60)	0.75 (0.49–1.15)	1.11 (0.82–1.50)
*ESR1*				
Model 1^a^	–	1.13 (0.48–2.60)	0.98 (0.69–1.39)	1.04 (0.78–1.40)
Model 2^b^	1.01 (0.82–1.25)	1.12 (0.48–2.60)	0.98 (0.69–1.39)	1.07 (0.80–1.40)
*PIK3CA*				
Model 1^a^	–	0.98 (0.66–1.40)	0.89 (0.72–1.10)	1.06 (0.89–1.30)
Model 2^b^	0.98 (0.86–1.11)	1.01 (0.68–1.50)	0.89 (0.72–1.10)	1.04 (0.87–1.20)
*GATA3*				
Model 1^a^	–	0.49 (0.29–0.82)**	0.80 (0.59–1.09)	0.69 (0.49–0.98)*
Model 2^b^	0.74 (0.60–0.91)**	0.42 (0.25–0.72)**	0.80 (0.59–1.09)	0.69 (0.48–0.97)*
*TP53*				
Model 1^a^	–	1.73 (1.17–2.56)**	2.20 (1.79–2.70)***	2.14 (1.75–2.62)***
Model 2^b^	2.05 (1.79–2.34)***	1.77 (1.20–2.63)**	2.20 (1.79–2.70)***	2.15 (1.75–2.64)***
*MAP3K1*				
Model 1^a^	–	0.24 (0.07–0.86)*	0.60 (0.35–1.03)	0.70 (0.49–1.00)*
Model 2^b^	0.65 (0.49–0.86)**	0.25 (0.07–0.89)*	0.60 (0.35–1.03)	0.70 (0.49–1.00)*
*ARID1A*				
Model 1^a^	–	1.19 (0.51–2.70)	0.75 (0.48–1.20)	0.97 (0.70–1.40)
Model 2^b^	0.92 (0.72–1.18)	1.19 (0.52–2.80)	0.75 (0.48–1.20)	0.97 (0.69–1.30)
*PTEN*				
Model 1^a^	–	2.04 (1.05–4.00)*	1.29 (0.92–1.82)	1.17 (0.86–1.60)
Model 2^b^	1.28 (1.03–1.58)*	2.11 (1.08–4.10)*	1.30 (0.92–1.82)	1.18 (0.86–1.60)
***CNVs***				
*ERBB2–AMP*				
Model 1^a^	–	0.21 (0.08–0.58)**	0.36 (0.22–0.58)***	0.62 (0.40–0.96)*
Model 2^b^	0.44 (0.33–0.59)***	0.21 (0.08–0.58)**	0.36 (0.22–0.58)***	0.63 (0.41–0.99)*
*MYC–AMP*				
Model 1^a^	–	1.06 (0.69–1.60)	1.55 (1.18–2.03)**	1.49 (1.13–2.00)**
Model 2^b^	1.45 (1.22–1.72)***	1.10 (0.71–1.70)	1.56 (1.19–2.05)**	1.57 (1.19–2.10)**
*RAD21–AMP*				
Model 1^a^	–	0.99 (0.62–1.60)	1.26 (0.93–1.71)	1.14 (0.85–1.50)
Model 2^b^	1.16 (0.96–1.41)	1.02 (0.64–1.60)	1.26 (0.93–1.71)	1.18 (0.87–1.60)
*ZNF217–AMP*				
Model 1^a^	–	0.69 (0.29–1.70)	0.89 (0.62–1.29)	0.85 (0.52–1.40)
Model 2^b^	0.91 (0.69–1.19)	0.68 (0.28–1.60)	0.89 (0.62–1.29)	0.84 (0.52–1.40)
*GATA3–AMP*				
Model 1^a^	–	0.96 (0.51–1.80)	0.96 (0.62–1.82)	1.31 (0.90–1.90)
Model 2^b^	1.10 (0.85–1.42)	0.96 (0.51–1.80)	0.96 (0.62–1.49)	1.35 (0.93–2.00)
*MYBL1–AMP*				
Model 1^a^	–	1.17 (0.59–2.30)	1.08 (0.71–1.63)	1.23 (0.85–1.80)
Model 2^b^	1.13 (0.88–1.46)	1.18 (0.59–2.40)	1.08 (0.71–1.63)	1.27 (0.87–1.90)
*CCND1–AMP*				
Model 1^a^	–	0.51 (0.32–0.81)**	1.00 (0.79–1.28)	1.22 (0.97–1.50)
Model 2^b^	1.03 (0.88–1.19)	0.47 (0.29–0.75)**	1.00 (0.79–1.28)	1.22 (0.97–1.50)
*EXT1–AMP*				
Model 1^a^	–	1.01 (0.59–1.70)	1.33 (0.93–1.90)	1.15 (0.80–1.70)
Model 2^b^	1.22 (0.98–1.53)	1.04 (0.61–1.80)	1.33 (0.93–1.90)	1.21 (0.84–1.70)
*FGFR1–AMP*				
Model 1^a^	–	1.51 (0.85–2.70)	1.13 (0.87–1.47)	1.35 (1.05–1.70)*
Model 2^b^	1.24 (1.05–1.47)*	1.51 (0.85–2.70)	1.13 (0.87–1.47)	1.37 (1.06–1.80)*
*MCL1–AMP*				
Model 1^a^	–	1.14 (0.61–2.10)	1.47 (0.97–2.23)	1.23 (0.82–1.80)
Model 2^b^	1.26 (0.98–1.64)	1.16 (0.63–2.20)	1.47 (0.97–2.24)	1.21 (0.81–1.80)

*y* years, *HR* hazard ratio, *MBC* metastatic breast cancer, *SNVs* single nucleotide variants, *CNVs* copy number variants, *AMP* amplification.

^a^Models test for one gene at a time and are adjusted for tumor subtype (HER2- luminal A, HER2- luminal B, HR + /HER2 + , HR-/HER2 + , HR-/HER2-), histology (invasive ductal, invasive lobular, mixed, other (including DCIS, micropapillary, mucinous, tubular, other) and unknown), TMB, initial stage of BC dx (de novo v. Stage 0-III), timepoint sampled for genetic sequencing (primary v. metastatic tumor) and race (White, Black, Asian, other, unknown), number of metastatic sites, and site of metastatic disease (bone, liver, lung, brain- all as yes/no). Individuals with unknown race (N = 54) or stage of initial diagnosis (N = 23) were excluded to allow for age-group comparisons.

^b^Adjusted for all covariates listed above plus continuous age at MBC diagnosis (years).

*p < 0.05, ** *p* < 0.01, *** *p* < 0.001.

In unadjusted models, SNVs in *TP53* and *PTEN* were associated with shorter OS for patients of all age groups (*TP53*: *p* < 0.001, *PTEN*: *p* < 0.01), while *PIK3CA, MAP3K1, and GATA3* mutations were associated with prolonged OS (*p* < 0.001 for all) (Fig. S5). Associations with OS were consistent for *TP53* and *MAP3K1* regardless of de novo vs. recurrent MBC status; however, associations were only significant for *GATA3, PIK3CA*, and *PTEN* among patients with rMBC (Fig. S6). In multivariable adjusted Cox models gene-survival associations were maintained for SNVs in *TP53* (hazard ratio, HzR [95% CI] = 2.05 [1.79-2.34], *p* < 0.001), *PTEN* (HzR [95% CI] = 1.28 [1.03-1.58], *p* < 0.05), *MAP3K1* (HzR [95% CI] = 0.65 [0.49-0.86], *p* < 0.01), and *GATA3* (HzR [95% CI] = 0.74 [0.60-0.91], *p* < 0.01) (Table 3). In crude models stratified by subtype, associations between *TP53* SNVs and OS were consistent across luminal A, luminal B, and TNBC subtypes (Fig. S7). Mutational frequencies for other significant genes were not large enough to assess significance in HER2+ and TNBC subtypes, though luminal A and B subtypes were compared. *PTEN* associations were not seen in either luminal subtype. MAP3K1 associations were consistent and significant for both luminal subtypes, and *GATA3* associations were only significant in luminal B patients (Fig. S8).

In general, age group at MBC diagnosis did not modify the associations between individual gene mutation status (CNV or SNV) and OS. One exception was *ERBB2* amplification; younger individuals with *ERBB2* amplification had a larger survival benefit compared to older individuals (p-interaction=0.03; *ERBB2-AMP* ≤ 40 years HzR [95% CI] = 0.21 [0.08–0.58] vs. >55 years HzR [95% CI] = 0.63 [0.41–0.99]). Other trends for age-group differences emerged, though low numbers of mutation carriers in some age groups limits comparisons. Improvement of OS observed with presence of *GATA3* SNVs was strongest among the youngest age-group (≤ 40 years *GATA3* SNV HzR [95% CI] = 0.42 [0.25–0.72], *p* < 0.01) and weaker in other age groups (though still significant in those >55 years). *CCND1*-amplifications were associated with longer OS in younger, but not older, patients (HzR [95% CI] ≤40 years = 0.47 [0.29-0.75], *p* < 0.01). The negative impact of harboring an SNV in *PTEN* on OS was much stronger in the youngest age group (HzR [95% CI] ≤40 years=2.11 [1.08-4.10], *p* < 0.05) compared to those >55 years at MBC diagnosis (HzR [95% CI] ≤40 years=1.18 [0.86–1.60]) (Table 3).

## Discussion

In this study of 2357 MBC patients, we found distinct tumor genomic profiles for those diagnosed ≤40 years vs. those diagnosed at later ages, expanding upon our previous comparison focused on older-onset MBC patients ( ≥ 70 years vs. <50 years)^19^. Mutational frequences differed by age group for *TP53, CDH1, and PIK3CA*^19^, which suggests that underlying differences in tumor biology of MBC patients exist across the age continuum. Our findings suggest that observed age-specific distribution of somatic alterations may contribute to survival differences in very young vs. older MBC patients. This highlights the importance of characterizing tumor genomics in MBC patients of all ages to provide better treatment and follow-up guidance.

Low TMB seen among the youngest group of patients aligns with previous studies^24,25^, and is consistent with known biological processes of aging, wherein DNA replication and repeated exposure to environmental carcinogens over the life course lead to accumulation of oncogenic mutations^26^. Interestingly, we found that age-related differences in TMB were more prominent for individuals with rMBC as opposed to those with dnMBC, and were present in nearly all tumor subtypes, despite known differences in TMB by molecular subtype^27^, suggesting that age influences TMB through alternative pathways. To our knowledge, this is the first study to show that, among HR + /HER2- MBC patients, age-related differences in TMB are driven by luminal B-like tumors, which are characterized by lower expression of HRs, higher expression of proliferative genes, more rapid progression, and reduced survival outcomes compared to luminal A tumors^28^. Further investigation is needed to understand why enhancements in age-related differences in TMB appear in subsets of patients with rMBC and with luminal B tumors as this may be informative for treatment with immunotherapy. Several studies have shown increased immune cell infiltration in tumors with higher TMB^29,30^ and increased survival for those treated with immune checkpoint inhibitors who have high TMB^31–33^. Thus, the lower TMB in younger rMBC patients suggests fewer potential candidates for immune checkpoint blockade in this patient group.

We found DNA amplification of *ERBB2, GATA3*, and *MYC* were more common among younger MBC patients. The differential frequency of *ERBB2* amplifications by age was significant even among patients with HER2+ tumors. While this may be due to lack of adjustment for aneuploidy, which is higher in younger patients, if this is a true association this is of interest given the proven success of anti-HER2 therapy in the metastatic disease setting, and known associations of *ERBB2* amplifications with OS^34^. Though this did not translate to age-related differences in OS in those with HER2+ tumors in our cohort, we were limited by relatively small numbers in this subset. Two small studies (N < 300), covering a broad age range (26-82 years), have separately reported high frequency of *GATA3* amplifications in triple-negative MBC^35^, and significant *GATA3* copy number gains for HER2-/HR+ and HER2 + MBC^36^. To our knowledge, amplifications in *GATA3* have not been widely recorded for younger vs. older MBC patients, as research surrounding this gene focuses on splice site and frameshift mutations^37^. Amplification of *MYC*, which plays a role in estrogen-induced proliferation as well as cellular differentiation, apoptosis, and metabolism^38^, has been commonly reported in overall breast cancer (15%)^39^ and MBC (13% in HER2-/ER+ tumors)^40^, aligning with the 11% frequency seen here. *MYC* amplification is associated with poor clinicopathologic features including high mitotic activity^41^ and 50% shorter OS^40^, consistent with the shortened survival seen in our study (HzR=1.45). *MYC* DNA amplification has been observed in the context of *BRCA1* germline or somatic inactivation^38^ and has been found to appear alongside *TP53* somatic mutations in early-stage breast cancer, at about 8% frequency^41,42^. Future studies exploring the connections between *BRCA1*, *MYC*, and *TP53* alterations may reveal distinguishing etiological processes in young MBC. Moreover, higher frequency of alterations in the *MYC* pathway genes in younger patients observed here supports further investigation into this pathway to advance the understanding of young MBC.

Mutations in *TP53* are estimated to be present in 30% of HR + MBC, 55% of HER2 + MBC, and >50-90% of triple-negative MBC, similar to frequencies seen in our study (28% HER2-/HR+ , 66% HER2+ , 83% TNBC)^43^. Here, alterations in *TP53* were consistently associated with a 2-fold shorter survival among patient groups, which is in line with previous studies of both dnMBC and rMBC patients^36,44,45^. Given the high mutational frequency, the observed shorter OS in patients with mutations, and the low clinical actionability at present^46^, finding effective strategies to target patients with tumors harboring *TP53* alterations remains a high priority in current research^43,47,48^.

Studies to date have not examined age-specific mutational distribution of *GATA3*, though this gene is known to be commonly mutated in advanced breast cancer^46^. Interestingly, though an age difference still exists in MBC, young age has less of an influence on *GATA3* mutational frequency in MBC vs. early-onset BC^20,21,49,50^. Our finding of longer survival among MBC patients with *GATA3* mutations supports the potential role of GATA3 as an inhibitor of metastatic spread, leading to more favorable prognosis^51–53^. However, *GATA3* has also been indicated as a tumor promoter^37,49,54^, possibly via stimulation of ER-alpha expression^55^ and, in turn, cell proliferation.

Older patients were more likely to have *CDH1* mutations in our study (≤ 40 years, 1% vs. >55 years, 18%), consistent with prior findings of early-stage breast cancer patients (≤ 40 years [*N* = 89]: 4% vs. >40 years [*N* = 949]: 18% vs. >60 years [*N* = 465]: 21%)^21^. While it is known that lobular subtypes are more common among older patients^56^, we found striking age-specific differences in CDH1 mutational frequency even when restricting the analysis to individuals with lobular MBC. This points to a potential biological difference between the lobular breast cancer arising in younger vs older women. Mutations in *CDH1* were not found to have an impact on survival in our study, indicating that *CDH1* may be more important in tumor initiation rather than progression.

Two key genes acting within the PI3K/AKT pathway, *PIK3CA* and *MAP3K1*, were more frequently mutated in older MBC compared to younger MBC patients in our study, aligning with age-related findings in early-stage breast cancer patients^21^. The PI3K/AKT pathway is essential in regulating cell proliferation and survival^57^, and therefore, mutations affecting this pathway are associated with hyperactivity and enhanced cell proliferation. Mutations in *PIK3CA* directly activate the signaling cascade, whereas *MAP3K1* and *PTEN* indirectly influence the PI3K/Akt pathway through negative regulation^58^. The association between an alteration in the PIK3-pathway and age highlights the importance of this pathway in providing insight to age-dependent tumor progression processes. Although *PTEN* mutations were not associated with age in this study, the presence of a *PTEN* mutation did confer a survival disadvantage for women of all ages, and this contrasts with the longer survival observed among those with *PIK3CA* and *MAP3K1* mutations. While a study among early-stage breast cancer patients showed similarly prolonged survival for those with *MAP3K1* mutations, our findings for *PTEN* and *PIK3CA* were not mirrored in early-stage disease^59^, necessitating further studies to confirm our results in MBC patients. PI3K pathway inhibition with agents like everolimus, alpelisib or inavolisib reverses resistance to anti-estrogen therapies in HR+ patients^60–63^, and as such, identifying these clinically actionable somatic mutations informs patient care decisions^46^. However, given that the frequency of PI3K pathway mutations is low in young MBC patients, novel drivers of tumor biology must be further studied so that we have more therapeutic options for young women with metastatic ER + /HER2- breast cancer.

Despite low frequency of *BRCA1/2* somatic mutations ( < 5%) in this population, somatic mutations in *BRCA1/2* were approximately two times as prevalent in younger-onset MBC tumors. Currently, there are now data demonstrating clinical benefit to PARP inhibitor therapy among those with MBC and tumors harboring somatic *BRCA* mutations^64^. Given that PARP inhibitors may be especially beneficial among the youngest MBC patients, it is important to prioritize tissue and blood-based methods to identify somatic BRCA mutations.

Irrespective of somatic mutation status, we found survival to be shorter among young recurrent MBC patients compared to older patients, while no survival differences were noted by age among those with dnMBC, suggesting potentially more rapid development of therapeutic resistance among younger women. Based on separate findings that (1) *TP53* mutations were more common in younger individuals and (2) shorter OS was associated with *TP53* mutations, it is plausible that age-specific distribution of somatic frequencies could contribute to survival differences among recurrent MBC patients, though further research is required. Moreover, larger studies should prioritize evaluating associations between gene mutations and survival by molecular subtype to refine clinical utility of this finding.

Given that large genomic databases such as TCGA and METABRIC consist of primary tumor samples, the key strength of this study is the large number of MBC patients (*N* = 2357) with tumor genomic sequencing. With 320 patients ≤40 years at MBC diagnosis, we were able to conduct genomic comparisons for the youngest group of MBC patients, which has not been possible in smaller studies to date. EMBRACE is a well-annotated clinical dataset, ensuring appropriate adjustment for tumor and patient factors when assessing both prevalence of genomic alterations and survival rates. Stratification of HR + /HER2- breast cancers by luminal A-like and luminal B-like subtypes enhanced our analysis, as these subtypes have not been extensively explored in MBC. However, it is important to consider that these subtypes were inferred, and misclassification may be present. While it is possible that some SNVs identified by OncoPanel could have been present in the germline, we accounted for this by conducting a sensitivity analysis excluding any individuals with known germline mutations, which did not reveal substantial changes in the results. Unfortunately, we did not have metastatic tumor samples directly available for OncoPanel sequencing for 766 of the 2,357 patients and relied on primary tumor genomic characterization for these patients. While sensitivity analysis restricted to metastatic tumor samples resulted in similar conclusions overall, likely due to identification of several truncal alterations in this dataset that are present in both primary and metastatic tumors, molecular subtype switching^65^ and evolution of tumor somatic alterations from primary to MBC^66,67^ may influence results. Given the complex and varied nature of metastatic treatment regimens, we did not adjust for treatments within our survival analyses. In addition to study design limitations, additional complexities in characterizing MBC tumor genomic profiles should be considered. It is known that genomic alterations present differently based on site of metastatic disease; for instance, brain metastases have been found to harbor a very distinct set of mutations^68^. We did not include separate assessment of age-related tumor genomic differences by site of metastases in order to maintain power to detect genomic alterations by age group in our study. With sufficient sample sizes, future examination of genomics within metastatic tumor subtypes may be beneficial to further improve the characterization age-related differences in MBC.

Overall, our study highlights the importance of tumor genomic profiling in MBC to understand age-specific etiological differences to inform treatment and surveillance in these patients in the hopes of improving both survival and quality of life. While our study shows clear differences in tumor profiles by age group in MBC patients, additional research is needed to understand the functional implications for tumorigenesis and development of recurrence and therapeutic resistance of the alterations that are differentially present in tumors of younger compared to older patients with MBC.

## Methods

### Cohort

The EMBRACE study is an ongoing prospective clinical cohort that has enrolled over 4,000 women with MBC who have been seen at DFCI since 2009. Patients are approached for consent to EMBRACE at the initial visit at DFCI and are approved under the Dana-Farber/Harvard Cancer Center (DF/HCC) institutional review board (IRB), studies #09-204 and/or #93-085. Patient demographics, primary tumor characteristics and treatments, metastatic tumor characteristics (including site and molecular subtyping if available), metastatic treatment regimens, and patient outcomes are collected. Survival follow-up is completed through medical record review, patient contact, and linkage to the National Death Index. In addition, if participants agree to tumor genomic sequencing for research (approved by DF/HCC IRB #11-104), targeted DNA sequencing is performed on metastatic and/or primary tumor samples. Patients who consented to EMBRACE, had available targeted DNA sequencing via OncoPanel in at least one primary or metastatic tumor sample, and were not missing age at MBC diagnosis, were included in this analytic cohort. All protocols were in accordance with the Declaration of Helsinki and approved by the DF/HCC IRB.

### Tumor Classification

Tumors were classified based on immunohistochemical (IHC) staining by molecular subtype: HR + /HER2- (classified as luminal A- or B-like, with luminal-B like defined by primary tumor grade=3 or progesterone receptor staining <10%), HR + /HER2 + , HR-/HER2 + , and HR-/HER2-. HR status was determined by estrogen receptor (ER)/progesterone receptor (PR) staining, with positivity considered ≥1% staining. HER2 was considered negative if rated 0 or 1+ by IHC, and positive if 3+ by IHC. In cases of equivocal HER2 tests (2 + ) fluorescence in situ hybridization ( ≥ 2 considered positive) was used to assign HER2 status, following ASCO/CAP guidelines^69^.

### OncoPanel Testing

Targeted tumor-only next-generation sequencing testing with OncoPanel was performed on formalin-fixed, paraffin-embedded tumor samples of primary and metastatic tumors following previously described methods^44,70^. Briefly, after confirming ≥20% tumor cellularity by histopathologic review, DNA was extracted and analyzed using a solution-phase Agilent SureSelect hybrid capture kit and an Illumina HiSeq 2500 sequencer. To pass quality control, mean target coverage of 187X unique, high quality, mapped reads per sample (range 50–844X; 50X minimum) was required. OncoPanel testing started in 2013, and patients with tumor samples that were sequenced between July 2013-December 2020 were included in this analysis to allow for >5 years of follow-up. Three different OncoPanel versions have been used over time, covering 275 gene coding regions (version 1, used until August 2014), 309 gene coding regions (version 2, used until October 2016), and 447 gene coding regions (version 3, used from October 2016 onward).

### Bioinformatics Pipeline

For this analysis, we used subtyping and genomic information from the earliest metastatic tumor sample available. If metastatic tumor samples were unavailable, the earliest primary sample was used. Somatic nucleotide variants were called with Mutect (v1.1.4) (RRID: SCR_000559) and insertion and deletions (indels) were called using the Genome Analysis Toolkit (GATK-version 1.6-5-g557da77) (RRID: SCR_001876). Common single nucleotide polymorphisms (SNPs) were detected and variants with a population frequency of >0.1% in the Exome Sequencing Project database (RRID: SCR_012761) were filtered as described previously^70^. Filtered SNPs were added back if they appeared in the COSMIC database (RRID: SCR_00260) more than twice, and then manual review determined their final inclusion, based on review of data demonstrating biological significance. This pipeline leads to exclusion of most germline variants, though some rare germline variants may remain. SNP and in-frame insertion and deletion variants were included if classified in OncoKB (RRID: SCR_014782) as oncogenic or likely oncogenic. Loss-of-function mutations were included if listed in OncoKB as tumor suppressor genes. As a result, VUS and benign/likely benign variants were not selected for profiling. CNVs were identified as amplifications if possessing gains with an average log_2_ ratio for a given interval ≥2, or if copy count >6, and as losses if possessing 2-copy deletions. Genes common to all three versions of OncoPanel (*N* = 239 genes and selected intronic regions of 30 genes)^44^ and altered in at least 1% of the cohort were included in main analyses (*n* = 32 SNVs and *n* = 51 amplifications or deletions). After filtering of SNVs and CNVs, TMB was estimated as the sum of exonic mutations per sample normalized by the size of the exonic bait-set of the panel used. Subtype-specific analyses also considered genes with alterations appearing at a frequency of ≥5% in each subtype.

### Statistical Analysis

Descriptive characteristics of cohort participants were summarized by means (standard deviation) and frequencies (%). Continuous and high v. low ( ≥ 10 v. <10 mutations/megabase)^71^ TMB and CNV and SNV variant frequencies were compared by age group at MBC diagnosis based on the definition for young-onset typically used in clinical practice (≤ 40 years) and the age distribution of the cohort (≤ 40, >40-55, >55 years) using Fisher’s exact test, overall and within tumor molecular subtypes. For overall analysis, SNVs or CNVs that appeared in ≥1% of the population were included. Within subtype-specific analysis, differences in SNVs or CNVs by age group were assessed if present at a frequency of ≥1% for specific subtypes.

Multivariable logistic regression models were used to test the odds of possessing individual gene mutations or CNVs by age group at MBC diagnosis (reference ≥55 years). Specific gene alterations, amplifications, and deletions were assessed if they were found to be significantly enriched or depleted (*p* < 0.05) in at least one molecular subtype (as determined by the Fisher’s exact test), and if they appeared at a frequency of >5% in the entire population. Adjustment factors included tumor molecular subtype (HR + /HER2- luminal A-like, HR + /HER2- luminal B-like, HER2 + , and HR-/HER2-); histology (invasive ductal [IDC]), ILC, mixed [IDC & ILC], other [inclusive of DCIS, micropapillary, mucinous, and tubular types], or unknown); race (White, Black, Asian, other, unknown); TMB; stage (dnMBC vs. rMBC); and primary v. metastatic sample type.

Kaplan-Meier curves, stratified by age group, assessed OS measured from time of MBC diagnosis by genetic mutation status within each age group. Multivariable Cox regression analysis was used to estimate HzRs and 95% CIs for OS by gene alteration status, considering each gene separately. All Cox models were adjusted for molecular subtype, TMB, histology, race, stage at initial diagnosis, and sample type as defined above, as well as age at metastatic diagnosis (years), number of metastatic sites, and site of metastatic disease (bone, liver, lung, brain). We further tested effect modification between gene alteration status and age group at MBC diagnosis using an interaction term in Cox models.

### Pathway Analysis

Genes were grouped into canonical signaling pathways based on several TCGA studies: RTK-Ras, PI3K, TP53, Cell-cycle, Myc, Notch, Wnt, TGF-β, NRF-2, and HIPPO^72^. Alteration frequency of oncogenic signaling pathways was defined based on presence of SNVs and CNVs in pathway genes with a known functional consequence (gain or loss of function). Analyses assessing the association between age at MBC diagnosis and frequency of oncogenic signaling pathway alterations was completed as above, using Fisher’s exact test.

### Sensitivity Analysis

All SNV, CNV, and pathway analyses were also conducted separately for individuals with metastatic tumor samples. To assess the contribution of germline variants that were not filtered in data processing steps in the OncoPanel data, patients who also underwent genetic testing for germline variants as part of their clinical care and were known to have P/LP germline variants were identified via cross-linking with a genetic database at DFCI. Logistic regression models and survival analyses were repeated among all participants known to be negative for any germline P/LP variants from this subset.

## Supplementary information

Supplementary Information

## Data Availability

Deidentified genomic data for 2100 samples in this cohort is available via AACR Project GENIE cBioPortal. These data are under restricted access and can be accessed when agreeing to the terms and conditions of GENIE use.
