# Supplementary material for: Tumor genomics in patients younger than 40 years of age with metastatic breast cancer
Source: NPJ Precis Oncol. 2026 Feb 26;10:144. doi: 10.1038/s41698-026-01333-0 (PMC13057355; doi:10.1038/s41698-026-01333-0)
Supplement: Supplementary file 1 — Supplementary Information [file 41698_2026_1333_MOESM1_ESM.docx]

**SUPPLEMENTAL TABLES and FIGURES**

**Tumor genomics in patients younger than 40 years of age with metastatic breast cancer**

**Supplemental Tables: 2**

**Supplemental Figures: 8**

| **Table S1**. Metastatic sites used for OncoPanel Sequencing, by age group at MBC diagnosis (N=1,572)^1^ | | | | |
| --- | --- | --- | --- | --- |
|  | Age group at metastatic diagnosis | | | |
| Site | All ages, N (%) | ≤40 y, N (%) | 41-55y, N (%) | >55 y, N (%) |
| Adrenal Gland(s) | 6 (0.4%) | 0 (0.0%) | 1 (0.1%) | 5 (0.3%) |
| Ascites | 7 (0.4%) | 0 (0.0%) | 0 (0.0%) | 7 (0.4%) |
| Axillary Lymph Node | 66 (4.2%) | 7 (0.4%) | 25 (1.6%) | 34 (2.2%) |
| Bone | 175 (11.1%) | 18 (1.1%) | 63 (4.0%) | 94 (6.0%) |
| Brain/CNS | 123 (7.8%) | 30 (1.9%) | 50 (3.2%) | 43 (2.7%) |
| Chest Wall | 108 (6.9%) | 10 (0.6%) | 40 (2.5%) | 58 (3.7%) |
| Contralateral Breast | 26 (1.7%) | 5 (0.3%) | 7 (0.4%) | 14 (0.9%) |
| Intra-abdominal | 36 (2.3%) | 3 (0.2%) | 10 (0.6%) | 23 (1.5%) |
| Ipsilateral Breast | 33 (2.1%) | 4 (0.3%) | 18 (1.1%) | 15 (1.0%) |
| Liver | 361 (23.0%) | 43 (2.7%) | 153 (9.7%) | 165 (10.5%) |
| Lung | 91 (5.8%) | 3 (0.2%) | 43 (2.7%) | 45 (2.9%) |
| Lymph Nodes, Distant | 103 (6.6%) | 6 (0.4%) | 42 (2.7%) | 55 (3.5%) |
| Lymph Nodes, Regional | 29 (1.8%) | 3 (0.2%) | 14 (0.9%) | 12 (0.8%) |
| Other, please specify | 5 (0.3%) | 1 (0.1%) | 1 (0.1%) | 3 (0.2%) |
| Ovary | 21 (1.3%) | 3 (0.2%) | 14 (0.9%) | 4 (0.3%) |
| Pericardium | 1 (0.1%) | 0 (0.0%) | 1 (0.1%) | 0 (0.0%) |
| Pleura | 34 (2.2%) | 4 (0.3%) | 16 (1.0%) | 14 (0.9%) |
| Pleural Effusion | 35 (2.2%) | 4 (0.3%) | 11 (0.7%) | 20 (1.3%) |
| Primary Breast | 240 (15.3%) | 49 (3.1%) | 85 (5.4%) | 106 (6.7%) |
| Skin (non-breast only) | 36 (2.3%) | 0 (0.0%) | 18 (1.1%) | 18 (1.1%) |
| Soft Tissue | 32 (2.0%) | 4 (0.3%) | 11 (0.7%) | 17 (1.1%) |
| ^1^ This includes only those with OncoPanel sequencing directly from metastatic tumor samples (N=1,591). N=19 patients with metastatic tumor sampling were listed as “unknown” for stage of diagnosis and were excluded here. | | | | |

| **Table S2**. Association between oncogenic and likely oncogenic tumor gene mutations and age group at MBC diagnosis, excluding 110 individuals with germline P/LPVs (N=2,198)^1^ | | | | |
| --- | --- | --- | --- | --- |
| Gene | Age group (y) at MBC diagnosis ^2^ | OR (95% CI)^3^ | p-value | q-value^4^ |
| *CDH1* | <=40 | 0.10 (0.03-0.31) | 8.4e-05*** | 3.0e-04*** |
|  | 40-55 | 0.54 (0.36-0.79) | 1.9e-03** | 4.7e-03** |
| *PIK3CA* | <=40 | 0.58 (0.42-0.81) | 1.5e-03** | 5.3e-03** |
|  | 40-55 | 0.86 (0.70-1.06) | ns | ns |
| *TP53* | <=40 | 1.85 (1.35-2.53) | 1.2e-04*** | 3.5e-04*** |
|  | 40-55 | 1.54 (1.23-1.93) | 1.5e-04*** | 4.0e-04*** |
| *GATA3* | <=40 | 1.35 (0.85-2.15) | ns | ns |
|  | 40-55 | 1.36 (0.97-1.90) | ns | ns |
| *ESR1* | <=40 | 0.41 (0.18-0.93) | 3.3e-02* | ns |
|  | 40-55 | 0.77 (0.52-1.12) | ns | ns |
| *MAP3K1* | <=40 | 0.55 (0.26-1.14) | ns | ns |
|  | 40-55 | 0.60 (0.39-0.93) | 2.3e-02* | ns |
| *PTEN* | <=40 | 0.63 (0.33-1.20) | ns | ns |
|  | 40-55 | 0.97 (0.67-1.42) | ns | ns |
| *ARID1A* | <=40 | 0.72 (0.35-1.45) | ns | ns |
|  | 40-55 | 0.95 (0.63-1.45) | ns | ns |
| MBC=metastatic breast cancer; P/LPV=pathogenic/likely pathogenic variants; y=years; OR=odds ratio; CI=confidence interval; ns=nonsignificant at α=0.05; * <0.05, ** <0.01, *** <0.001  1 Genes selected if mutational frequency >5% in total population and if mutation was significantly enriched or depleted (p<0.05) in at least one tumor molecular subtype. N total excludes those missing subtype or stage.  2 Reference age group is >55y at MBC diagnosis  3 All models adjusted for tumor subtype (HER2- luminal A, HER2- luminal B, HR+/HER2+, HR-/HER2+, HR-/HER2), histology (invasive ductal, invasive lobular, mixed, other (including DCIS, micropapillary, mucinous, tubular), unknown), TMB, initial stage of BC (*de novo* v. Stage 0-III), timepoint sampled for genetic sequencing (primary v. metastatic tumor) and race (White, Black, Asian, other, unknown)  4 FDR-adjusted p-value | | | | |

**Figure S1**. Tumor Mutational Burden among patients with inferred HR+/HER2- luminal A vs. luminal B tumors. Metastatic samples (or primary tumor if a metastatic sample was not available) were included in the analysis. Tumors were defined as luminal B-like if progesterone staining was <10% in the metastatic or primary sample (depending on which was available for subtyping) and histologic grade of the primary tumor was 3.

P-value significance: *: p<0.05, **: p<0.01, ***: p<0.001, ****: p<0.0001

**
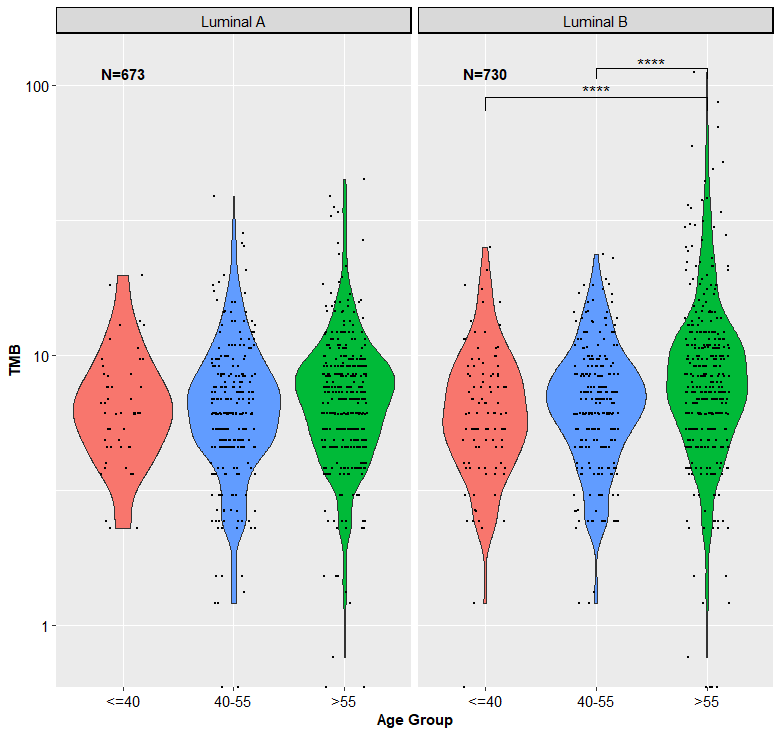
**

**Figure S2**. Frequency of gene alterations by age at MBC diagnosis by sample type sequenced: (A) metastatic tumor and (B) primary tumor. Genes included if alterations appeared at ≥5% frequency in overall sample (primary + metastatic samples) and were present in both primary and metastatic tumors.


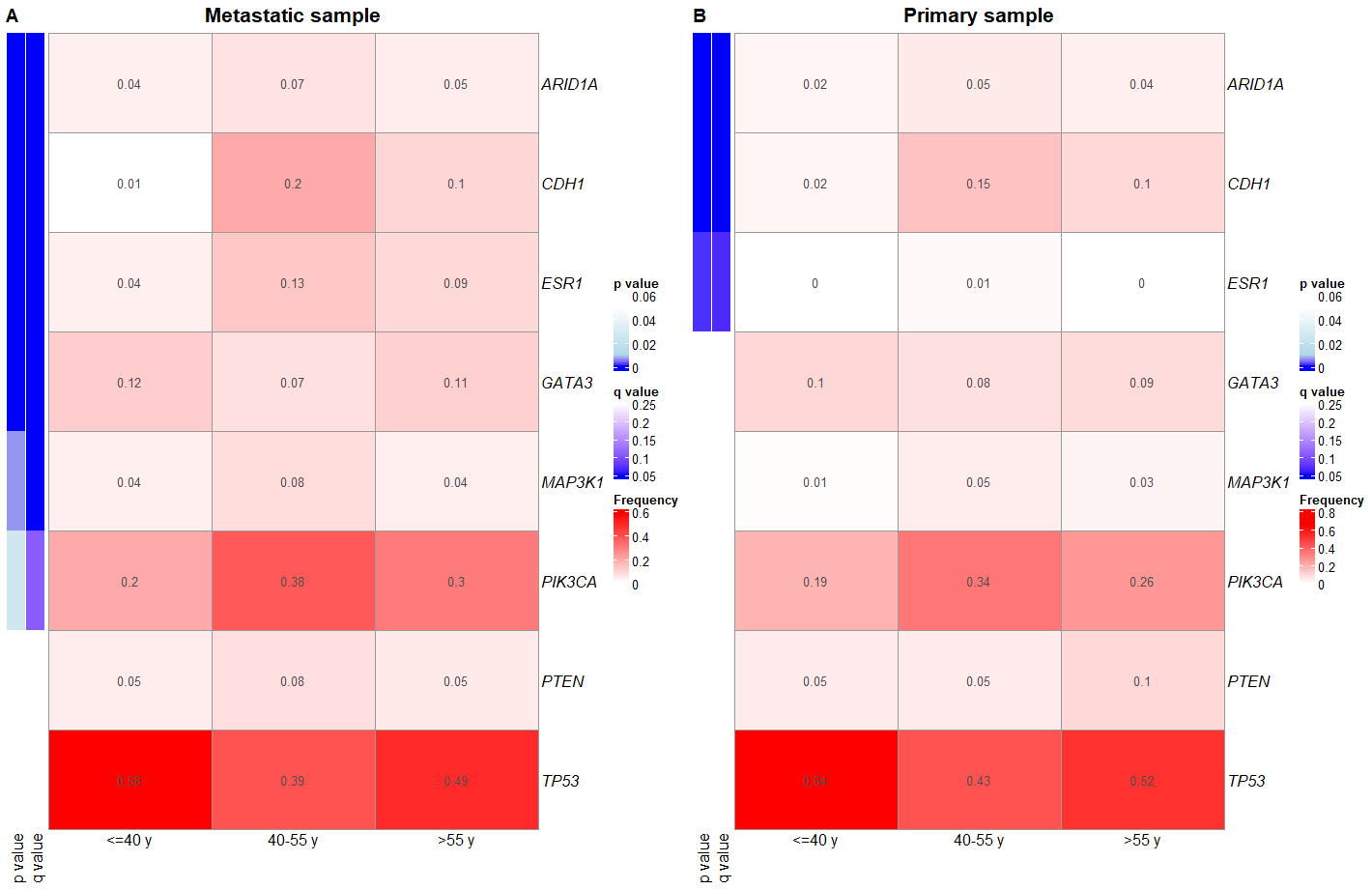


**Figure S3.** K-M plots for OS by age group at MBC diagnosis among individuals with Stage IV *de novo* MBC for (A) HR+/HER2- (B) HR+/HER2+ (C) HR-/HER2+, and (D) TNBC subtypes. P-values presented are for the log-rank test comparing individuals with vs. without gene mutations.

**
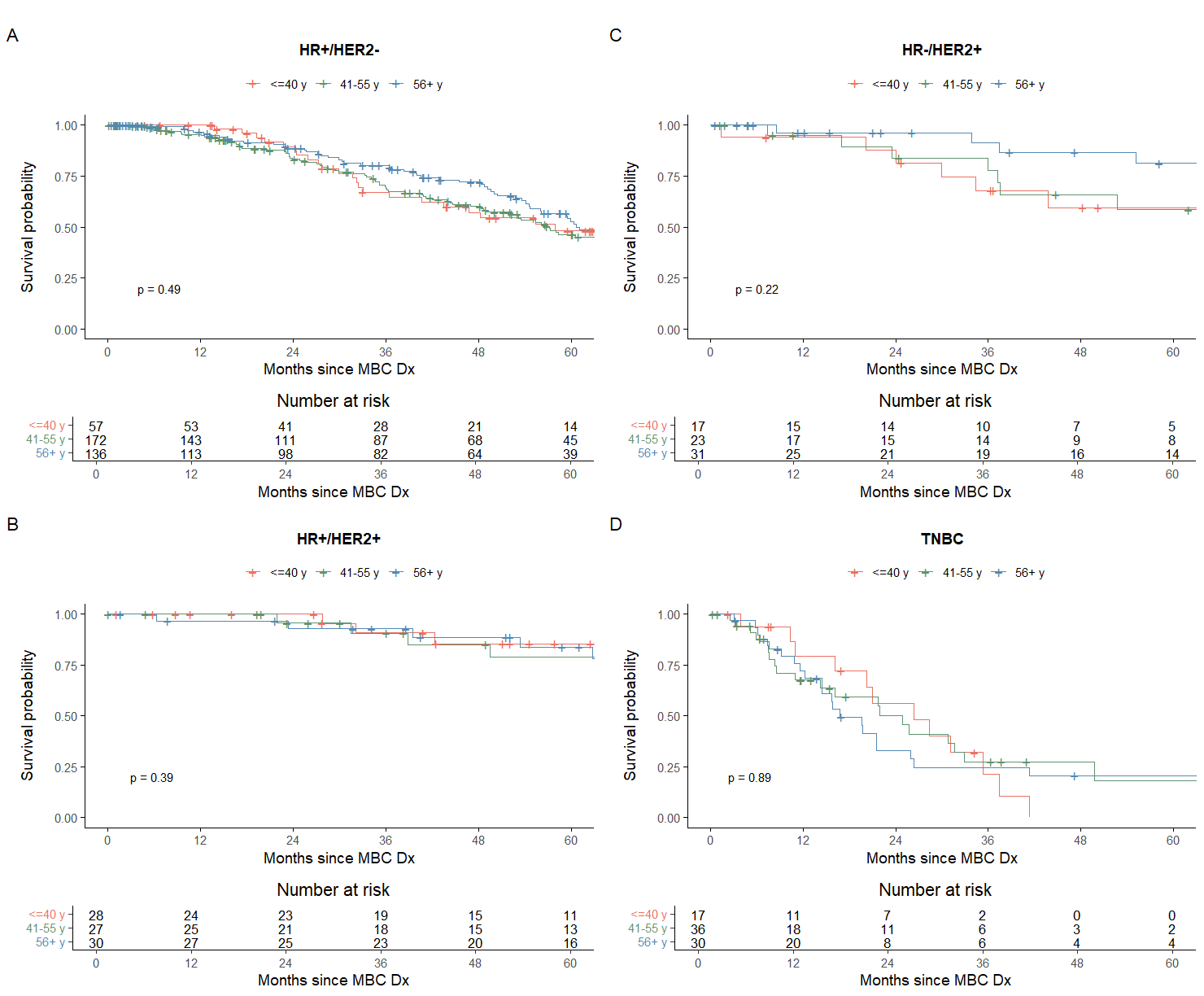
**

**Figure S4**. K-M plots for OS by mutational status of selected statistically significant gene amplifications among all patients (A) *ERBB2*-AMP (N=334), (B) *MYC*-AMP (N=257), (C) *FGFR1* (N=265). P-values presented are for the log-rank test comparing individuals with vs. without amplifications in genes.


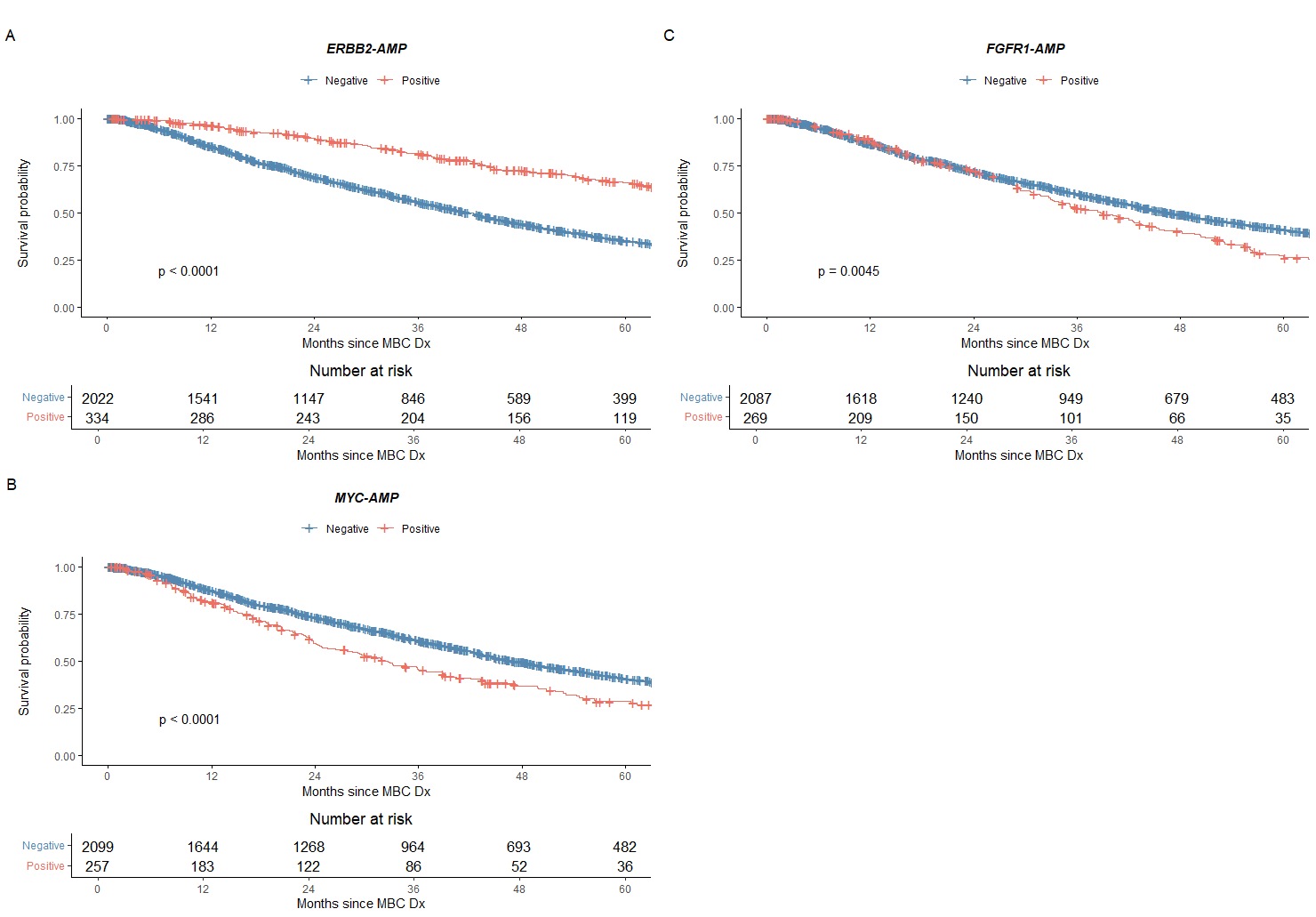


**Figure S5.** K-M plots for OS by mutational status (SNV) of statistically significant genes among all patients (N=2,357): (A) *TP53* (N=1,1106 mutated), (B) *PTEN* (N=151 mutated), (C) *GATA3* (N=212 mutated), (D) *MAP3K1* (N=122 mutated), (E) *PIK3CA* (N=740 mutated). P-values presented are for the log-rank test comparing individuals with vs. without SNV mutations in genes.


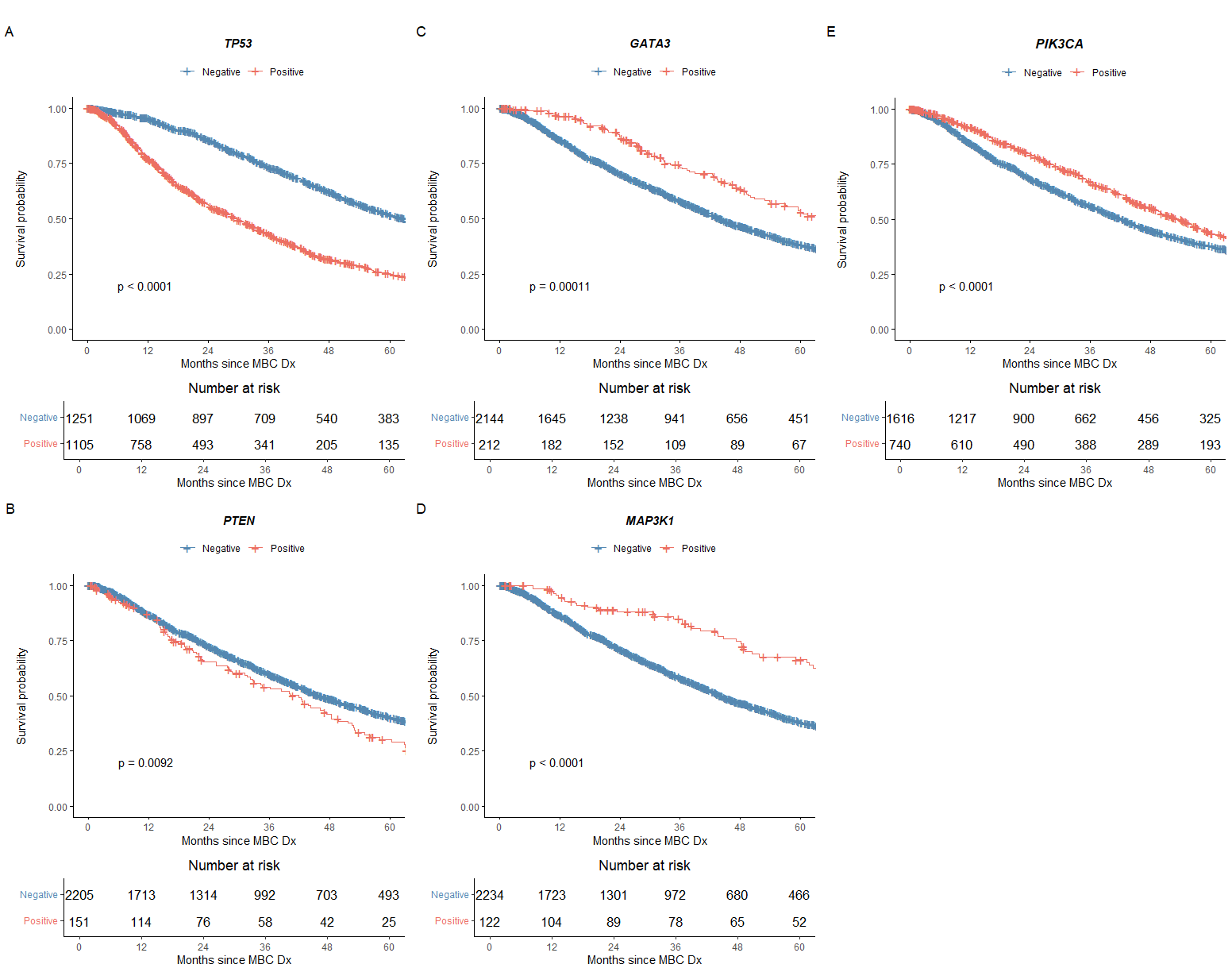


**Figure S6.** K-M plots for OS by mutational status by recurrent or *de novo* MBC status: (A) *GATA3*, recurrent MBC (N altered/N WT=145/1582) (B) *GATA3, de novo* MBC (N altered/N WT=64/543) (C) *PIK3CA,* recurrent MBC (N altered/N WT=537/1190), (D) *PIK3CA, de novo* MBC (N altered/N WT=193/414), (E) PTEN, recurrent MBC (N altered/N WT=122/1605) (F) PTEN, *de novo* MBC (N altered/N WT=29/578). P-values presented are for the log-rank test comparing individuals with vs. without SNV mutations in genes.

**
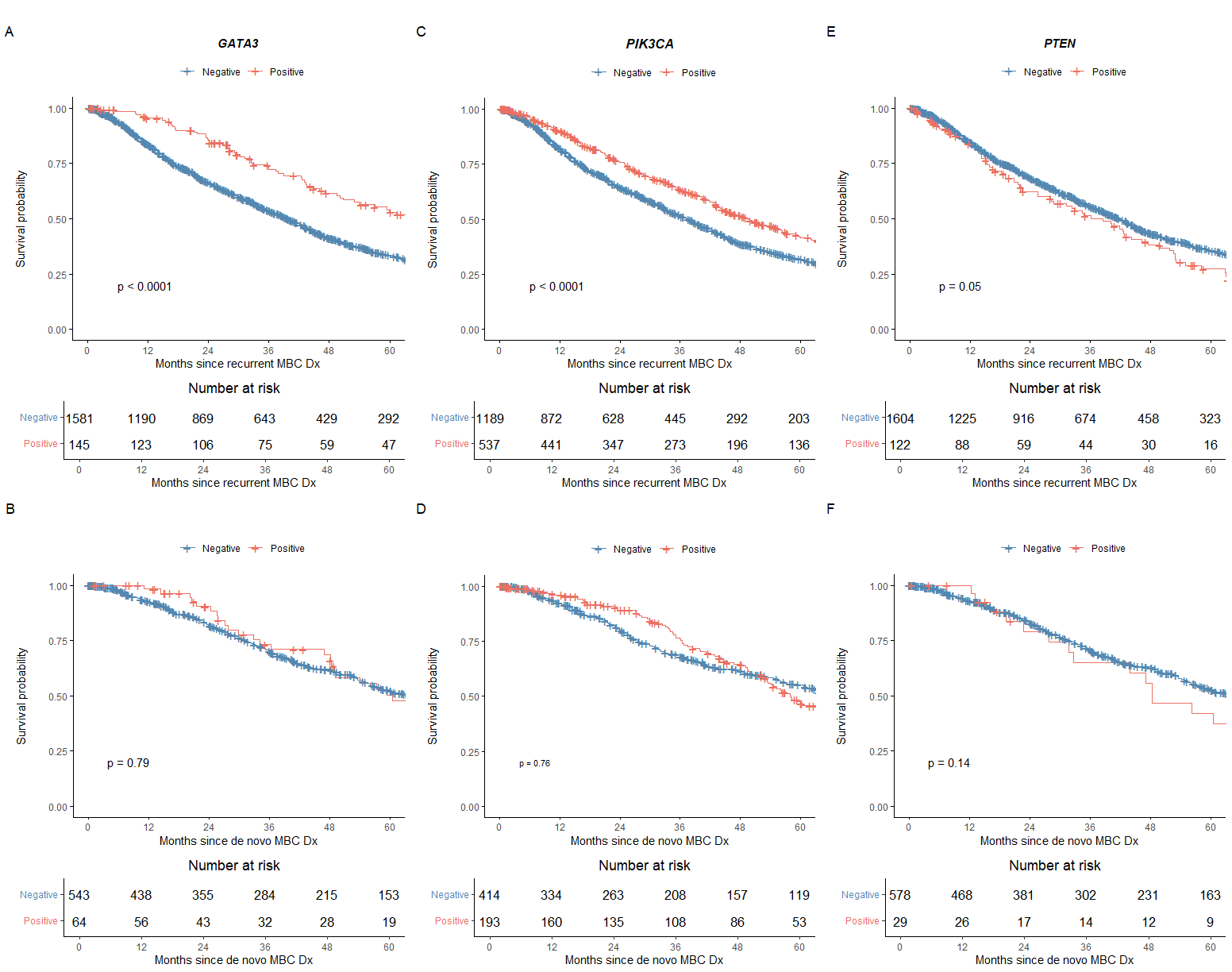
**

**Figure S7.** K-M plots for OS by *TP53* mutational status in recurrent MBC patients by subtype: (A) Luminal A (B) Luminal B (C) HER2+/HR+, (D) HER2+/HR+, (E) TNBC*.* P-values presented are for the log-rank test comparing individuals with vs. without SNV mutations in genes.

**
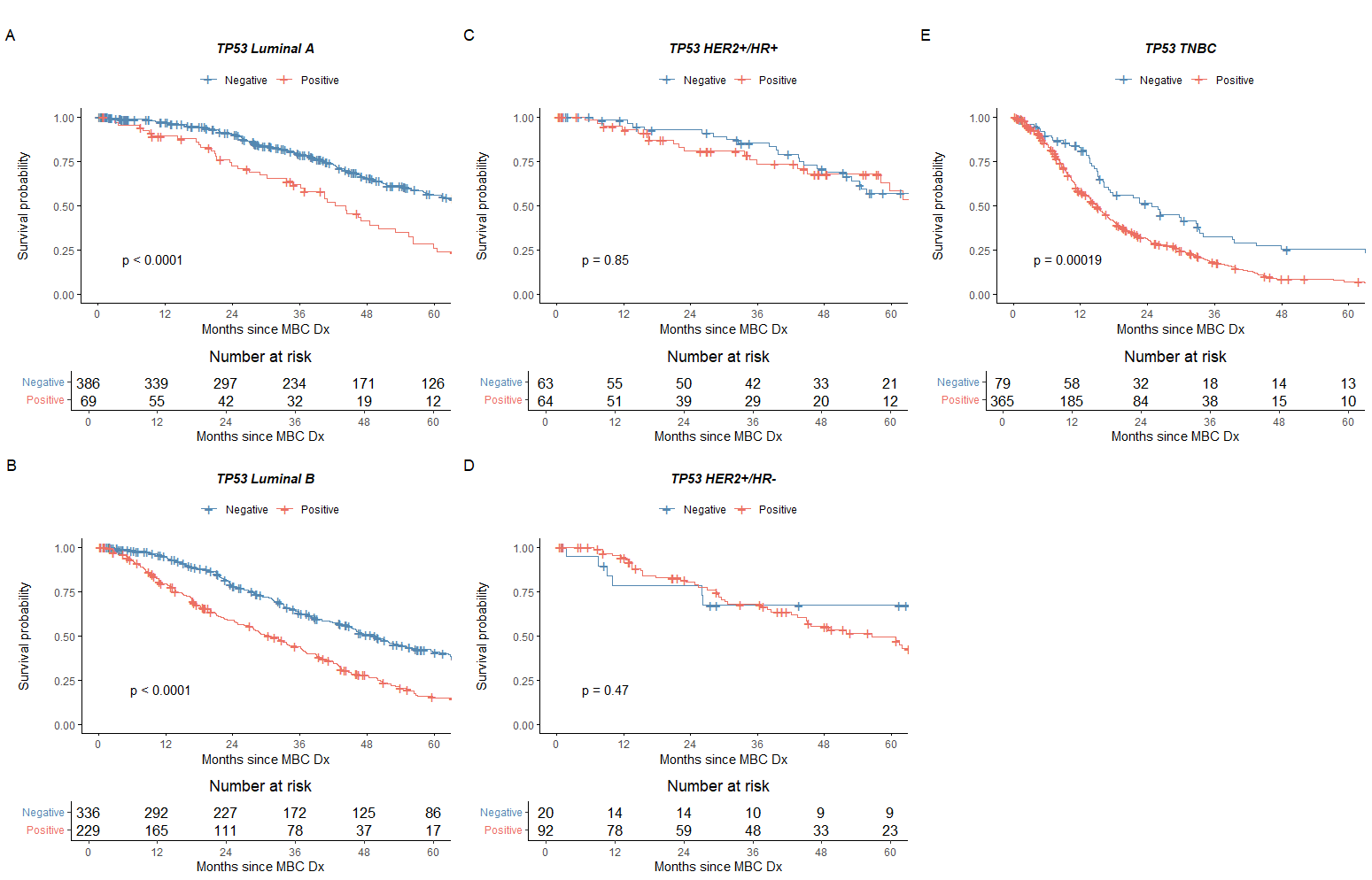
**

**Figure S8.** K-M plots for OS by gene mutational status in recurrent MBC patients by luminal-like subtype: (A) *PTEN* Luminal A (B) *PTEN* Luminal B (C) *GATA3* luminal A (D) *GATA3* luminal B (E) *MAP3K1* luminal A (F) *MAP3K1* luminal B. P-values presented are for the log-rank test comparing individuals with vs. without SNV mutations in genes.

**
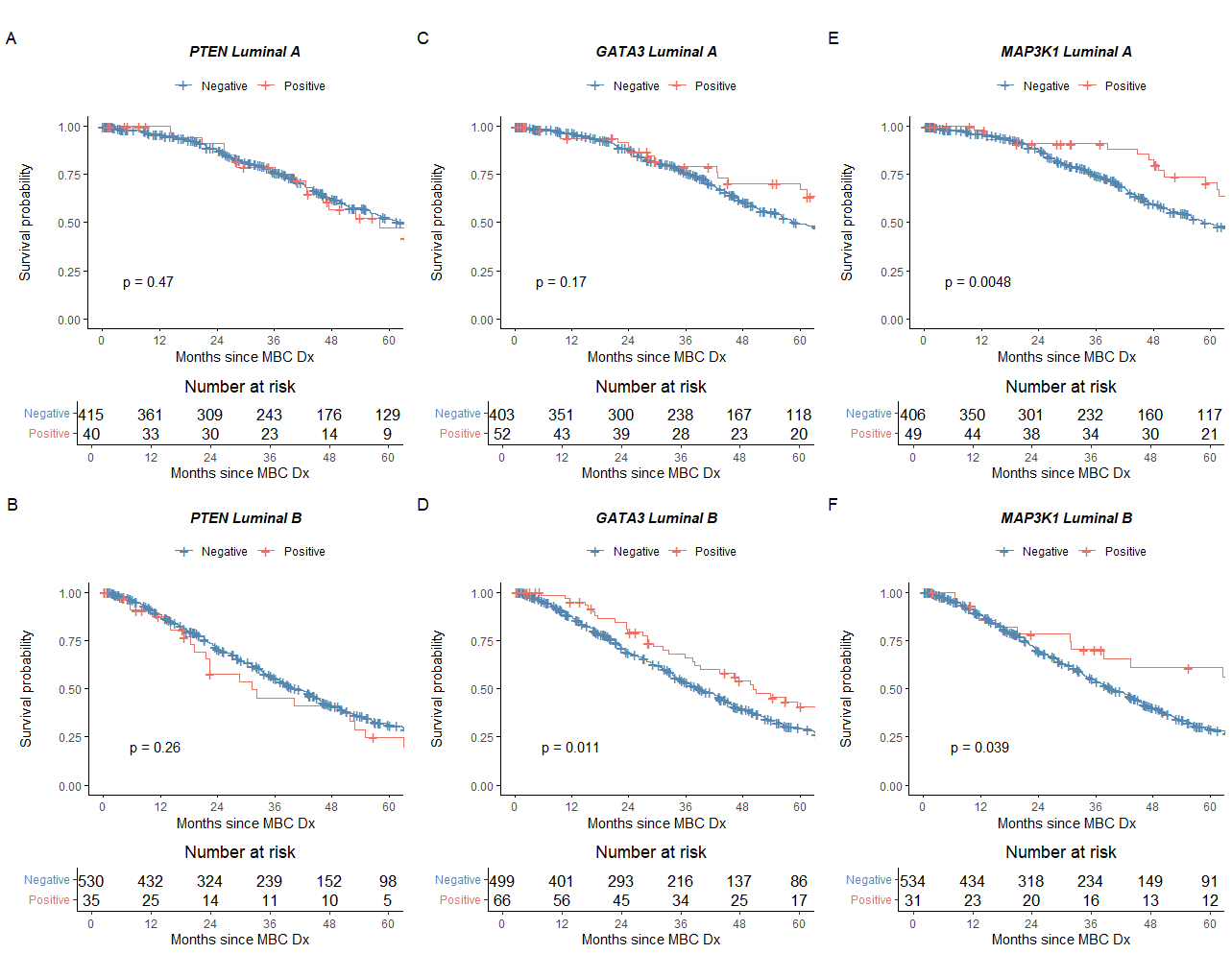
**
